# Improving low-temperature activity and thermostability of exo-inulinase InuAGN25 on the basis of increasing rigidity of the terminus and flexibility of the catalytic domain

**DOI:** 10.1080/21655979.2020.1837476

**Published:** 2020-10-31

**Authors:** Rui Zhang, Limei He, Jidong Shen, Ying Miao, Xianghua Tang, Qian Wu, Junpei Zhou, Zunxi Huang

**Affiliations:** a Engineering Research Center of Sustainable Development and Utilization of Biomass Energy, Ministry of Education, Yunnan Normal University, Kunming, People’s Republic of China; b College of Life Sciences, Yunnan Normal University, Kunming, People’s Republic of China; c Key Laboratory of Yunnan for Biomass Energy and Biotechnology of Environment, Kunming, Yunnan, People’s Republic of China

**Keywords:** Enzyme, biochemical property, structure, mechanism, mutagenesis

## Abstract

Enzymes displaying high activity at low temperatures and good thermostability are attracting attention in many studies. However, improving low-temperature activity along with the thermostability of enzymes remains challenging. In this study, the mutant Mut8S, including eight sites (N61E, K156R, P236E, T243K, D268E, T277D, Q390K, and R409D) mutated from the exo-inulinase InuAGN25, was designed on the basis of increasing the number of salt bridges through comparison between the low-temperature-active InuAGN25 and thermophilic exo-inulinases. The recombinant Mut8S, which was expressed in *Escherichia coli*, was digested by human rhinovirus 3 C protease to remove the amino acid fusion sequence at N-terminus, producing RfsMut8S. Compared with wild-type RfsMInuAGN25, the mutant RfsMut8S showed (1) lower root mean square deviation values, (2) lower root mean square fluctuation (RMSF) values of residues in six regions of the N and C termini but higher RMSF values in five regions of the catalytic pocket, (3) higher activity at 0–40°C, and (4) better thermostability at 50°C. This study proposes a way to increase low-temperature activity along with a thermostability improvement of exo-inulinase on the basis of increasing the rigidity of the terminus and the flexibility of the catalytic domain. These findings may prove useful in formulating rational designs for increasing the thermal performance of enzymes.

## Graphical abstract

**Figure:**
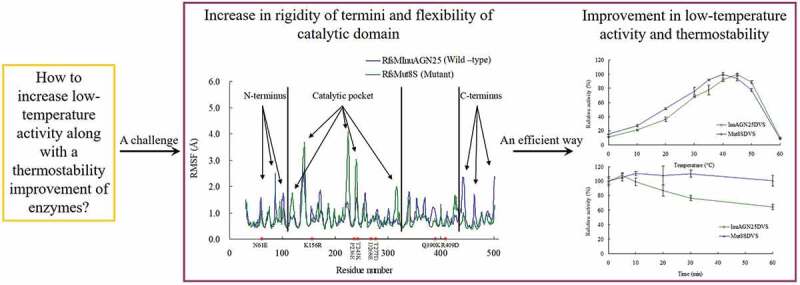

## 1. Introduction

Jerusalem artichoke (*Helianthus tuberosus*) is a promising biomass species with numerous advantageous characteristics, such as low-input cultivation, a high growth rate, and good anti-frost, anti-drought, anti-pest, and anti-disease properties [1]. The fleshy roots of the Jerusalem artichoke contain an impressive amount (12–19%) of inulin, which is a β-D-(2→1)-fructan linked to a terminal sucrose residue [2]. It is easy to extract raw inulin from the inulin-rich feedstock using the conventional water extraction method [2].

One important application of inulin is to produce fructose, which possesses functional properties of maintaining human health and technical advantages of various food and beverage additives [2]. Conventionally, starch is used to produce fructose *via* either acid hydrolysis or complex enzymatic reactions which involve α-amylase, amyloglucosidase, and glucoisomerase. There are a few disadvantages associated with the two conventional methods, such as low product yield, formation of byproducts, and additional purification and refining costs [2]. A single-step enzymatic method has been developed by using exo-inulinases to hydrolyze inulin, with a much high conversion efficiency of 90–95% [2]. Exo-inulinases are members of glycoside hydrolase (GH) family 32 and are designated EC 3.2.1.80, functioning as hydrolyzing terminal 2,1-linked β-D-fructose residues.

Thermal adaptations of enzymes are attracting much attention. On the one hand, thermostability is a critical enzymatic property, due to the high temperatures that occur during some industrial processes and the long-term storage requirement of enzymes. For example, inulin is extracted from Jerusalem artichoke using the conventional water extraction method at the temperature of 60°C considering that the solubility of inulin increases along with temperature rises in water [3]. Therefore, improvement in thermostability of exo-inulinases is meaningful to avoid thermal denaturation of enzymes during the inulin hydrolysis process and long-term storage. On the other hand, enzymes adapted to low temperatures (lower than 25°C) are also favorable, with the advantages of a low heating cost and convenient reaction environments, because low temperature environments cover more than 80% of the Earth’s biosphere [4].

A salt bridge is an electrostatic interaction and contributes greatly to global or local rigidity of the structure of enzyme. Usually, an increase in the salt bridge number, especially in the form of a salt bridge network, leads to an improvement in the thermostability of enzymes [5,6]. For example, Jonsdottir et al. demonstrated that the salt bridge network formed by three salt bridges, R259–D17, D17–R12, and R12–D183, within the N-terminal tail of aqualysin I remarkably contributed to its thermostability [7]. In the previous study, the salt bridge network formed by three salt bridges, K5–D8, D8–K12, and K12–D10, within the N-terminal tail of the mutant RfsMutNGln23Δ3, is considered to be one of the important reasons for the observed thermostability improvement [8].

Enzyme stability and activity is usually negatively correlated, because rigid tertiary structures hamper the activity of thermostable enzymes at low temperatures and flexible tertiary structures lead to poor thermostability of enzymes at high temperatures [9]. The phenomenon is known as the activity–stability trade-off of enzymes [9]. To date, improving low-temperature activity along with the thermostability of enzymes remains challenging.

Owing to the remarkably positive effect of salt bridges on the thermostability of enzymes, in this study we tried to introduce additional salt bridges within the structure of wild-type exo-inulinase InuAGN25 through site mutagenesis. This study aimed to improve both the low-temperature activity and the thermostability of an exo-inulinase on the basis of increasing rigidity of the terminus and flexibility of the catalytic domain.

## 2. Materials and methods

### 2.1. Chemicals, vectors, and strains

All reagents, purchased from commercial suppliers, were of analytical grade, including but not limited to human rhinovirus 3 C protease (HRV 3 C protease; TaKaRa, Otsu, Japan) and inulin from dahlia tubers (Sigma-Aldrich, St. Louis, MO, USA). The vector *p*ET-28a(+) and strain *E. coli* BL21 (DE3) used for heterologous expression were supplied by Synbio Technologies (Suzhou, China).

Previously, Zhou et al. isolated *Sphingobacterium* sp. GN25 from feces of *Grus nigricollis* and deposited the strain in the China General Microbiological Culture Collection Center under CGMCC 1.10975 [10]. A novel low-temperature-active exo-inulinase, InuAGN25 (accession no. AGC01503), which can hydrolyze Jerusalem artichoke tuber powder solution to produce fructose at 0°C and 10°C, was identified from the strain based on the molecular activity strategy [10].

### 2.2. Selection of mutagenesis site

Multiple amino acid sequence alignment and salt bridge prediction were carried out using Clustal X [11] and VMD [12], respectively. The tertiary structures of enzymes were predicted using the Swiss-Model platform (http://swissmodel.expasy.org/) and visualized using Discovery Studio v2.5 software (Accelrys, San Diego, CA, USA). To remove steric clashes, the structures were optimized by energy minimization using YASARA software (www.yasara.org).

Each molecular dynamics (MD) simulation of the wild-type enzyme and its mutant were performed using the AMBER 14 simulation package [13], and each system was repeated twice and established using the standard AMBER force field ff99SB [14]. The simulated system was immersed in a dodecahedral periodic box of TIP3P water molecules which extended 1.0 nm from the protein atoms [15]. Sodium or chloride ions were added to neutralize the charge. Before the MD simulations, energy minimization was carried out for the water molecules/ions via 1000 steps, the side chains of the enzyme via 20,000 steps, and the whole system *via* 4000 steps. After energy minimization, each system was simulated for 20 ns at 323 K, 1.0 bar with a time step of 2 fs, using the SHAKE algorithm [16] to constrain bonds that contained hydrogen atoms and the particle-mesh Ewald method [17] to treat long-range electrostatic interactions.

### 2.3. Construction of expression plasmid

Recombinant InuAGN25 (rInuAGN25), including a fusion sequence of 18 amino acid residues (derived from vector) at the N-terminus and mature InuAGN25 (MInuAGN25; the predicted signal peptide consisting of M1 to A22 was removed) was expressed previously in *E. coli* using *p*EASY-E1 as a vector [10]. Because the terminal fusion sequence can affect thermostability of enzymes [18], the expression plasmid for recombinant MInuAGN25 was reconstructed previously to insert the HRV 3 C protease recognition site, LEVLFQGP, into the N-terminus and the stop codon of TAA into the C-terminus, using *p*ET-28a(+) as a vector [8]. To avoid thermal denaturation of low-temperature-active enzymes during the digestion process, HRV 3 C protease is often used to remove fusion sequence from recombinant enzymes, considering that the protease displays high specificity as well as activity at low temperatures [19,20].

The mutant enzyme, designated as Mut8S, includes 8 sites mutated from MInuAGN25. To improve the readability of the manuscript, the amino acid residues of Mut8S were numbered from 23. In other words, the amino acid residue positions of Mut8S were in accordance with that of InuAGN25. Therefore, the mutation sites were N61E, K156R, P236E, T243K, D268E, T277D, Q390K, and R409D.

The recombinant mutant enzyme, designed as HHMut8S, contained the fusion amino acid sequence MGSSHHHHHHSSGLVPRGSHMASMTGGQQMGRGSEFLEVLFQ (approximately 4.6 kDa) at the N-terminus. The nucleic acid sequence encoding HHMut8S was synthesized by Synbio Technologies (Suzhou, China) with the addition of the restriction enzyme site of *Nco*I at the 5ʹ terminus, as well as the stop codon of TAA and the restriction enzyme site of *Xho*I at the 3ʹ terminus. After digestion with *Nco*I and *Sac*I, the sequences encoding HHMut8S and *p*ET-28a(+) were ligated using T4 DNA ligase. The nucleic acid sequence of mutant plasmid, termed his_6_-hrv-mut8S-p28, was confirmed by DNA sequencing (Tsingke, Beijing, China).

### 2.4. Recombinant enzyme expression and extraction

The mutant plasmid his_6_-hrv-mut8S-p28 was transformed into *E. coli* BL21 (DE3), according to the classic heat shock method. When the *E. coli* culture in Luria–Bertani medium (supplemented with 100 μg mL^−1^ kanamycin) reached an OD_600 nm_ of approximately 0.7, a final concentration of 0.7 mM IPTG was added to the culture to induce recombinant enzyme expression at 20°C for approximately 20 h. Cells were harvested by centrifugation and extracted by sonication on ice, as with rInuAGN25 [10].

### 2.5. Fusion sequence removal and purification of enzyme

HHMut8S was purified using the immobilized metal affinity chromatography method, the same as that of rInuAGN25 [10]. Then, HHMut8S was digested with HRV 3 C protease at 4°C for around 16 h to remove the fusion sequence. After loading the digestion products onto nickel-NTA agarose gel columns, (a) the cleaved fusion sequence and the protease were bound to the agarose; and (b) HHMut8S without the fusion sequence, designated as RfsMut8S, was eluted. The elution containing RfsMut8S was dialyzed three times against 1 L of McIlvaine buffer (pH 7.0) using a dialysis membrane with a molecular weight cutoff of 14 kDa. The fusion sequence removal and purity of purified RfsMut8S were identified by sodium dodecyl sulfate-polyacrylamide gel electrophoresis (SDS-PAGE).

### 2.6. Characterization of enzymes

Enzymatic properties of purified RfsMut8S were characterized using the classic 3,5-dinitrosalicylic acid (DNS) method with 0.5% (w/v) inulin as substrate. Half of the reaction system described previously [10] was employed: 450 μl of inulin solution was hydrolyzed by 50 μl of enzyme solution for 10 min, and then, the reaction was stopped by 750 μl of DNS reagent. The activity was calculated on the basis of absorption data measured at 540 nm, defining the amount of enzyme releasing 1 μmol of fructose per minute as one unit of activity.

The pH and temperature optima, as well as pH and thermal stabilities, of purified RfsMut8S were determined in triplicate as with RfsMInuAGN25 [8]. Briefly, the optimal pH of purified RfsMut8S was determined at 37°C; the optimal temperature of the enzyme was determined in pH 6.0 McIlvaine buffer; pH stability of the enzyme was determined by measuring the residual activity at 37°C in pH 6.0 McIlvaine buffer after incubating the enzyme at 20°C for 1 h without a substrate in the pH range of 3.0 to 11.0; thermostability of the enzyme was determined by measuring the residual activity at 37°C in pH 6.0 McIlvaine buffer after incubating these enzymes at 50°C for 5–60 min without a substrate.

Activation energies (*E*_a_) for catalytic reactions of wild-type RfsMInuAGN25 and its mutant RfsMut8S toward inulin were calculated on the basis of the Arrhenius plots method, as described previously [21]. *E*_a_ = −slope*R, where R is the universal gas constant (8.314 J K^−1^ mol^−1^) and slope was calculated using ln of velocity on the ordinate versus 1/T in Kelvin on the abscissa. The catalytic reactions were carried out with the purified enzymes using inulin as substrate at temperatures varying from 0°C to 35°C.

Considering that GH 32 includes inulinases functioning with exo- and endo-hydrolysis of inulin, 0.5% inulin (w/v) was hydrolyzed by RfsMut8S at 37°C, pH 6.0, for 4 h, and then the action mode of the inulinase was identified by visualizing the hydrolysis products with the use of the thin layer chromatography (TLC) method, as described previously [10]. Briefly, the TLC plate (silica gel G) was developed at room temperature using n-butanol/acetic acid/water at a volume ratio of 2:1:1, and then, saccharides were visualized using aniline–diphenylamine–phosphoric acid–acetone reagent.

## 3. Results

### 3.1. Selection of mutagenesis site

Previously, the homology model of exo-inulinase InuAGN25 was built successfully using the crystal structure of 1Y4W as a template, and structural comparison suggested that InuAGN25 has fewer predicted salt bridges than mesophilic, thermophilic, and hyperthermophilic exo-inulinases [10]. The predicted salt bridge decrease is proposed as one of the major factors accounting for the low temperature adaptation of InuAGN25 [10]. In this study, strong salt bridges (distance cutoff: 3.2 Å) were analyzed, as shown in Table 1. InuAGN25 had nine salt bridges; however, the hyperthermophilic exo-inulinase BfrA-Tm from *Thermotoga maritima* MSB8 (accession no. CAA04518 or PDB ID 1W2T) [22] had 24 salt bridges, the thermophilic exo-inulinase Inu-Aa from *Aspergillus awamori* var. 2250 (accession no. CAC44220 or PDB ID 1Y4W) [23] had 13 salt bridges, and the thermophilic exo-inulinase InuA-Gs from *Geobacillus stearothermophilus* KP1289 (accession no. BAC45010) [24] had 12 salt bridges.

**Table 1.** Salt bridges (distance cutoff: 3.2 Å) of GH 32 thermophilic exo-inulinases, InuAGN25, and Mut8S

Enzyme	Salt bridges
BfrA-Tm	24: **D52–K27**^1^, D53–H56, D67–K118, D68–H71, **D201–R196**^2^, D234–R281, D318–R304, D386–H7, E69–R37, E103–R137, E130–K156, E190–R137, **E219–K200**^3^, E286–K291, E297–R250, E308–K305, E325–R302, E325–R382, **E329–R380**^5^, E344–R337, E367–R262, E367–R369, E391–R281, E391–H7
Inu-Aa	13: D23–R486, D77–H80, D219–K200, **D292–K257**^4^, D311–R450, D485–H31, E82–K84, E99–R91, **E197–K247**^2^, E241–R188, **E283–K257**^3^, E490–R354, E490–H31
InuA-Gs	12: D149–R151, D199–R230, **D253–K219**^4^, D267–R404, **D394–K375**^5^, D437–H14, **E34–R129**^1^, E203–R151, **E209–K218**^2^, E364–R433, E442–R312, E442–H14
InuAGN25	9: D87–H90, D176–R178, D289–R419, D383–R381, D453–H41, E217–K214, E230–R178, E458–R331, E458–H41
Mut8S	13: **D59–R156**, D87–H90, D176–R178, D289–R419, **D301–K243**, D383–R381, D453–H41, **E61–R156**, E217–K214, E230–R178, **E268–K244**, E458–R331, E458–H41

**Figure 1.**
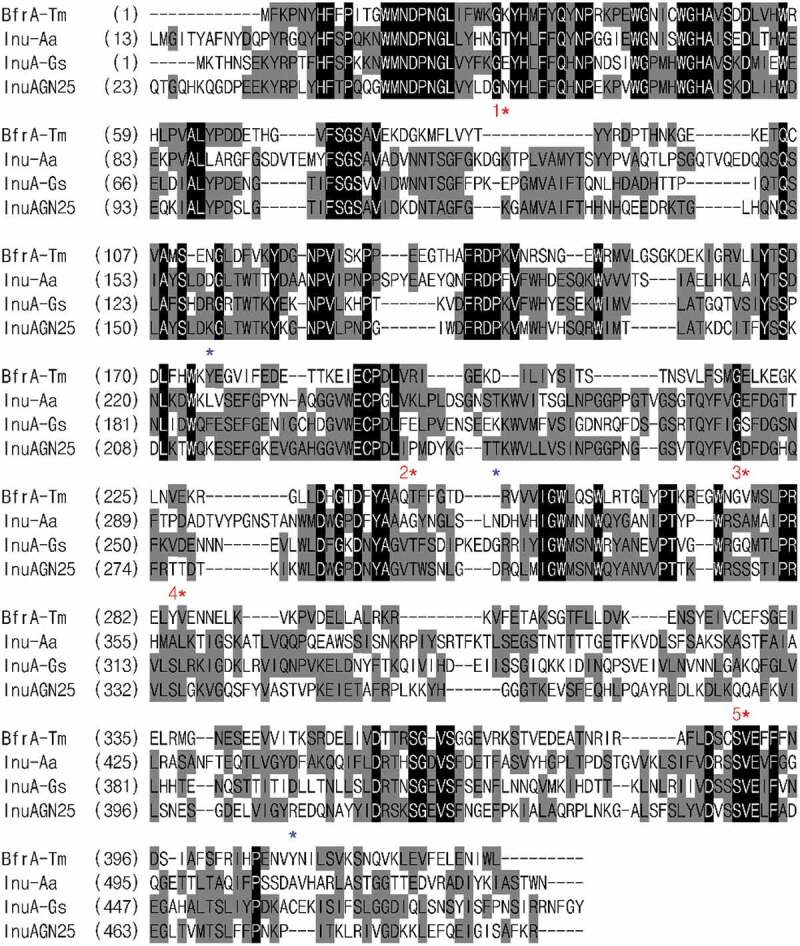
Amino acid sequence alignment of InuAGN25 with GH 32 thermophilic exo-inulinases

Numbers 1, 2, 3, 4, and 5 indicate the relative hotspots involved in salt bridge formation corresponding to the red asterisks shown in Figure 1.

Red font indicates 4 salt bridges observed in Mut8S other than InuAGN25.

BfrA-Tm: the hyperthermophilic exo-inulinase from *Thermotoga. maritima* MSB8 (accession no. CAA04518 or PDB ID 1W2T) [22]; Inu-Aa: the thermophilic exo-inulinase from *Aspergillus awamori* var. 2250 (accession no. CAC44220 or PDB ID 1Y4W) [23]; and InuA-Gs: the thermophilic exo-inulinase from *Geobacillus stearothermophilus* KP1289 (accession no. BAC45010) [24].

To find out the relative hotspots that are involved in forming salt bridges, the multiple amino acid sequence alignment of InuAGN25 with thermophilic and hyperthermophilic exo-inulinases was performed (Figure 1). As shown in Figure 1 and Table 1, five relative hotspots were found, considering that two or three salt bridges were observed in the three thermophilic exo-inulinases at each of these spots. Furthermore, three amino acid residue positions were also identified to coordinate the formation of salt bridges (Figure 1). Therefore, Mut8S containing mutation sites of N61E, K156R, P236E, T243K, D268E, T277D, Q390K, and R409D were designed.

BfrA-Tm: the hyperthermophilic exo-inulinase from T. maritima MSB8 (accession no. CAA04518 or PDB ID 1W2T) [22]; Inu-Aa: the thermophilic exo-inulinase from A. awamori var. 2250 (accession no. CAC44220 or PDB ID 1Y4W) [23]; and InuA-Gs: the thermophilic exo-inulinase from G. stearothermophilus KP1289 (accession no. BAC45010) [24]. Identical residues are shaded in black and conserved residues are shaded in gray. The asterisks indicate site-directed mutagenesis positions needed for salt bridge formation. Red asterisks indicate five relative hotspots involved in salt bridge formation, and blue asterisks indicate three positions coordinated the formation of salt bridges.

**Figure 2.**
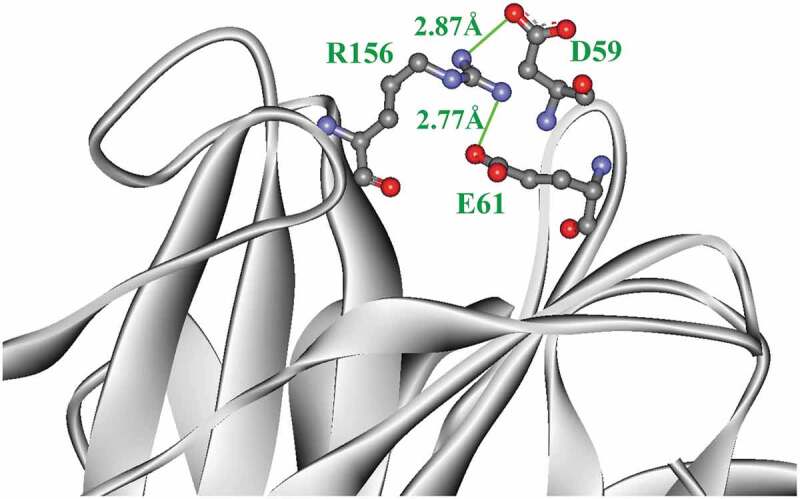
The salt bridge network (green) formed within the N-terminal tail of Mut8S

Mut8S had four salt bridges more than InuAGN25 (Table 1). Two salt bridges, D59–R156 and E61–R156, formed a salt bridge network within the N-terminus of Mut8S (Figure 2). However, that salt bridge network was not observed in InuAGN25. Meanwhile, these increased salt bridges in Mut8S did not break the salt bridge network formed by the three salt bridges, D453–H41, E458–H41, and E458–R331, within both the N-terminus and the C-terminus (Table 1).

Amino acid residues involved in salt bridges are shown in ball and stick form.

**Figure 3.**
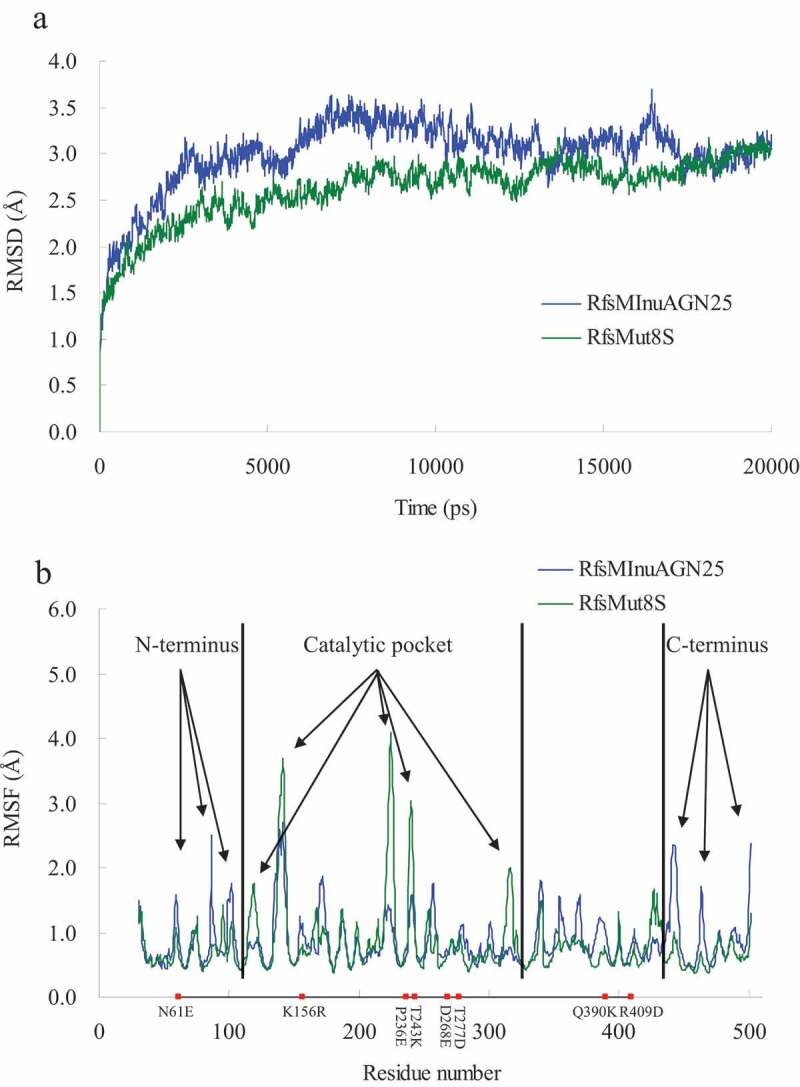
MD analysis of wild-type RfsMInuAGN25 and its mutant RfsMut8S at 323 K

To avoid the effects of the amino acid fusion sequence at the N-terminus on thermostability, MD simulations were carried out for RfsMInuAGN25 and its mutant RfsMut8S at 323 K for 20 ns. Generally, RfsMut8S had lower root mean square deviation (RMSD) values than wild-type RfsMInuAGN25 (Figure 3(a)), suggesting that the structure of RfsMut8S is more stable than that of RfsMInuAGN25. The local plasticity of wild-type RfsMInuAGN25 and the mutant RfsMut8S were evaluated through root mean square fluctuation (RMSF) values of Cα atoms calculated using MD simulation data of the final 8 ns with respect to the starting structures (Figure 3(b)). Generally, three large differences in RMSF curves were observed between RfsMInuAGN25 and RfsMut8S (Figure 3(b)). RMSF values of residues in three regions of the N-terminus of RfsMInuAGN25 were higher than those of RfsMut8S (Figure 3(b)). RMSF values of residues in three regions of the C-terminus of RfsMInuAGN25 were also higher than those of RfsMut8S (Figure 3(b)). However, RMSF values of residues in five regions of the catalytic pocket of RfsMInuAGN25 were lower than those of RfsMut8S (Figure 3(b)). These RMSF results suggested that the N-terminus and C-terminus of RfsMut8S were more rigid than those of RfsMInuAGN25, whereas the catalytic pocket of RfsMut8S was more flexible than that of RfsMInuAGN25.

(a) RMSD values during a 20-ns molecular dynamics simulation of the enzymes. (b) RMSF values of residues from the enzymes. Arrows indicate the flexibility changes at the local regions. Red dots indicate the amino acid residues selected for site mutagenesis.

### 3.2. Expression and purification of mutant enzyme

**Figure 4.**
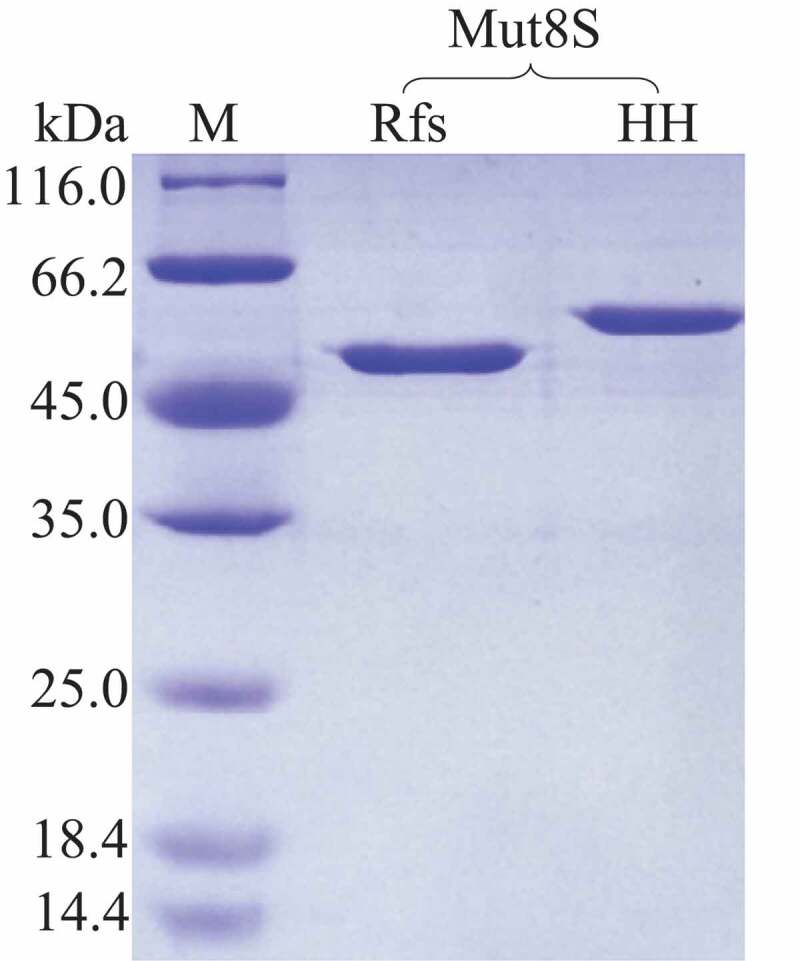
SDS-PAGE analysis

The mutant plasmid his_6_-hrv-mut8S-p28 was successfully constructed and transformed into *E. coli* BL21 (DE3). The His-tagged enzyme HHMut8S was expressed inside the host cells and purified using the immobilized-metal affinity chromatography method. After being incubated with the HRV 3 °C protease, the fused amino acid sequence (with a molecular weight of approximately 4.6 kDa) was cleaved from HHMut8S (Figure 4). The RfsMut8S in the digestion mixture was purified by affinity chromatography and protein purity reached the electrophoretically pure (Figure 4).

M, protein molecular weight marker; HH, purified HHMut8S; Rfs, purified RfsMut8S.

### 3.3. Properties of mutant enzyme

**Figure 5.**
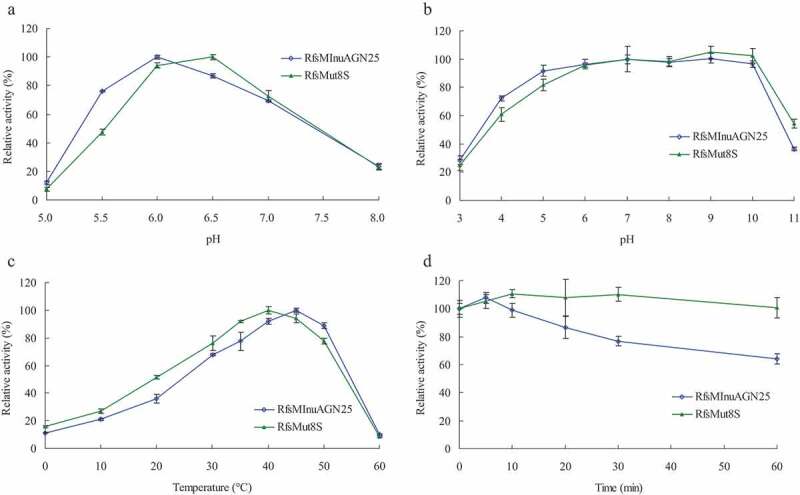
Effects of pH and temperature on purified wild-type RfsMInuAGN25 and its mutant RfsMut8S

The pH-dependent activity and stability of wild-type RfsMInuAGN25 have been characterized previously [8]. The optimal pH for enzymatic activity of purified RfsMInuAGN25 and RfsMut8S were pH 6.0 and 6.5 (McIlvaine buffer), respectively, when determining the activity at 37°C (Figure 5(a)). RfsMInuAGN25 and RfsMut8S showed similar curves of pH stability, retaining more than 60% residual activity after the incubation of these enzymes at 20°C for 1 h without a substrate at the pH range of 4.0 to 10.0 (pH 3.0–8.0 McIlvaine buffer and pH 9.0–10.0 0.1 M glycine–NaOH buffer; Figure 5(b)).

(a) pH-dependent activity. (b) pH-dependent stability. (c) Temperature-dependent activity. (d) Temperature-dependent stability. Error bars represent the means ± SD (*n* = 3). Enzymatic properties of wild-type RfsMInuAGN25 have been characterized previously [8].

The temperature-dependent activity and stability of wild-type RfsMInuAGN25 have been characterized previously [8]. The optimal temperatures for enzymatic activity of purified RfsMInuAGN25 and RfsMut8S were 45°C and 40°C, respectively, when determining the activity in pH 6.0 McIlvaine buffer (Figure 5(c)). RfsMut8S showed 5–15% activity higher than RfsMInuAGN25 when reactions happened at 0–35°C, but 11% activity lower than RfsMInuAGN25 when reactions happened at 50°C (Figure 5(c)). Thermostability differences between RfsMInuAGN25 and RfsMut8S were obvious. RfsMut8S was stable at 50°C for up to 60 min in pH 6.0 McIlvaine buffer without a substrate, whereas the residual activity of RfsMInuAGN25 reduced from 108% to 64% with the increase of incubation time from 5 min to 60 min (Figure 5(d)).

**Figure 6.**
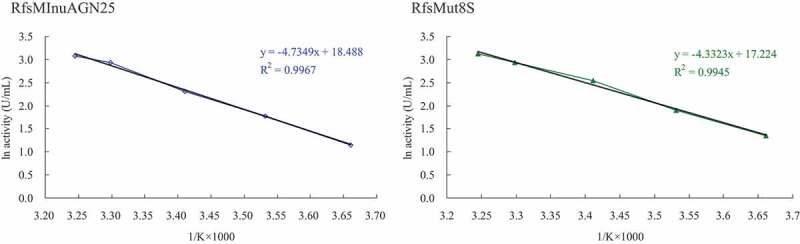
Arrhenius plots for the determination of *E*_a_ for inulin hydrolysis by wild-type RfsMInuAGN25 and its mutant RfsMut8S

On the basis of the Arrhenius plots (Figure 6), the *E*_a_ values for catalytic reactions of wild-type RfsMInuAGN25 and its mutant RfsMut8S toward inulin were 39.4 and 36.0 kJ mol^−1^, respectively.

**Figure 7.**
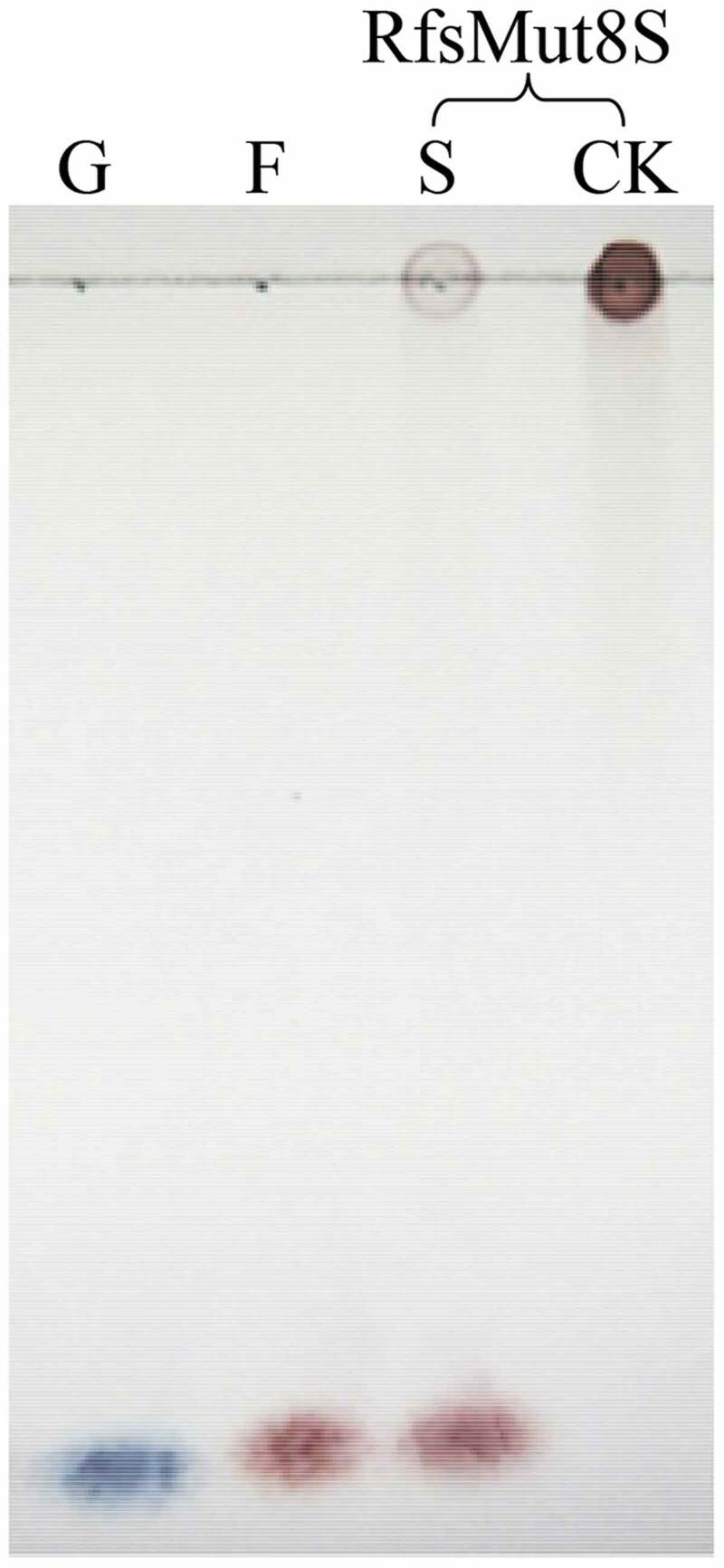
TLC analysis. G, 1.0% (w/v) glucose; F, 1.0% (w/v) fructose; S and CK, inulin with the active and inactivated enzymes, respectively

According to TLC analysis, RfsMut8S hydrolyzed inulin to produce fructose (Figure 7), suggesting that site mutation of the eight amino acid residues did not change the exo-action characteristic of the enzyme.

## 4. Discussion

Site mutagenesis results indicated that the mutation of N61E, K156R, T243K, and D268E led to the formation of the two salt bridges D301–K243 and E268–K244, as well as one salt bridge network consisting of the two salt bridges E61–R156 and D59–R156. These intraprotein interactions may enhance the global structural rigidity of the mutant RfsMut8S. Therefore, further RMSD analysis showed that the mutant RfsMut8S had lower RMSD values than wild-type RfsMInuAGN25.

RMSF analysis indicated that both the N-terminus and C-terminus of RfsMut8S were more rigid than those of RfsMInuAGN25. The formation of the salt bridge network by the two salt bridges E61–R156 and D59–R156 may contribute to the local rigidity of the N-terminus within the mutant RfsMut8S. It is unclear how the mutation of the eight amino acid residues affects the local rigidity of the C-terminus within the mutant RfsMut8S.

The effects of N and C termini of exo-inulinases on thermostability and structural rigidity have seldom been reported [8]. Most previous studies aimed to reveal the effects of conserved amino acid residues on exo-inulinase activity using the site mutagenesis method, such as the mutation of D189 within the conserved motif R-D-P, revealing the substrate recognition function of D189 [25]. N and C termini, as well as loops, are believed to prefer to unfold first during thermal denaturation of proteins [26]. Increasing the local structural rigidity by lowering the RMSF values of N and C termini is an effective way to improve the thermostability of enzymes [27–29].

In addition to the thermostability improvement, the comparison of the temperature-dependent activity curves and *E*_a_ values between wild-type RfsMInuAGN25 and its mutant RfsMut8S indicated that the low-temperature activity of the mutant was increased. This may be owing to the increase of RMSF in the five regions of the catalytic pocket. The mutation of P236E is directly responsible for the increased RMSF in one of the five regions, considering that proline residues allow harder local conformational changes [30] and that glutamic acid residues usually have a more pronounced average hydrophilicity and a higher absolute net charge [29].

It is difficult to overcome the activity–stability trade-off of enzymes during mutagenesis [31]. For example, deletion of the Ω-loop fragment ^74^YGSDVT^79^ from *Aspergillus niger* exo-inulinase resulted in a 12°C decrease in optimum temperature and thermostability loss at 60°C [32]. Substituting the histidine residue from ^189^AELH^192^ of the *A. niger* exo-inulinase with alanine residue resulted in a 5°C decrease in optimum temperature and thermostability loss at 60°C [33]. The optimum temperature and thermostability of *Kluyveromyces marxianus* exo-inulinase were simultaneously enhanced when the enzyme was fused to the inulin-binding module from the N-terminal region of *Bacillus macerans* cycloinulinooligosaccharide fructanotransferase [34]. Yu and Dalby found a way to counteract the enzyme activity–stability trade-off on the basis of dynamics correlations with the flexible active-site regions in a mutant of *E. coli* transketolase [31]. Lenz et al. chose the amino acid residues with low conservation scores at the entrance of the active pocket as hotspots for mutagenesis, and then the increased activity of *Caldicellulosiruptor saccharolyticus* β-glucosidase at lower temperatures without loss of stability was observed [35]. In this study, the low-temperature activity of the exo-inulinase was increased with the improvement of thermostability on the basis of increasing the rigidity of the N and C termini and the flexibility of the catalytic domain.

## 5. Conclusions

The eight-site-mutant Mut8S was designed on the basis of salt bridge comparison between wild-type InuAGN25 and thermophilic exo-inulinases. Four salt bridges, two of which formed a salt bridge network within the N-terminus, were observed only in Mut8S. Mut8S was expressed in *E. coli* and the recombinant Mut8S was digested by human rhinovirus 3 C protease to remove the amino acid fusion sequence at the N-terminus, producing RfsMut8S. Compared with wild-type RfsMInuAGN25, the mutant RfsMut8S showed significantly improved thermostability at 50°C, agreeing with the lower RMSF values in the six regions of the N and C termini and the lower RMSD values. Meanwhile, RfsMut8S showed significantly improved low-temperature activity, agreeing with the higher RMSF values in the five regions of the catalytic pocket. This study shows that the low-temperature activity of exo-inulinases can be increased, along with an improvement in thermostability, if the rigidity of the terminus and the flexibility of the catalytic domain are simultaneously increased. Further studies on single-site mutations and multi-site combination mutations of the eight residues from InuAGN25 may be useful to confirm the effects of each residue and related mechanisms.

## Abbreviations

DNS: 3,5-Dinitrosalicylic acid
*E*_a_: Activation energy
GH: Glycoside hydrolase family
HRV: Human rhinovirus
IPTG: Isopropyl-β-d-1-thiogalactopyranoside
MD: Molecular dynamics
RMSD: Root mean square deviation
RMSF: Root mean square fluctuation
SDS-PAGE: Sodium dodecyl sulfate-polyacrylamide gel electrophoresis
TLC: Thin-layer chromatography
InuAGN25: Exo-inulinase from *Sphingobacterium* sp. GN25
MInuAGN25: Mature InuAGN25 without the predicted signal peptide consisting of Met1 to Ala22
rInuAGN25: Recombinant MInuAGN25 expressed in *E. coli* using *p*EASY-E1 as vector
HHMInuAGN25: Recombinant MInuAGN25 with amino acid fusion sequence MGSSHHHHHHSSGLVPRGSHMASMTGGQQMGRGSEFLEVLFQ (containing a His_6_ tag and HRV 3C protease recognition site) at the N-terminus, expressed in *E. coli* using *p*ET-28a(+) as a vector
RfsMInuAGN25: Removal of amino acid fusion sequence from HHMInuAGN25 by HRV 3C protease
Mut8S: The mutant enzyme including 8 sites mutated from MInuAGN25. The amino acid residues of Mut8S were numbered from twenty-three. The mutation sites were N61E, K156R, P236E, T243K, D268E, T277D, Q390K, and R409D.
HHMut8S: Recombinant Mut8S with amino acid fusion sequence MGSSHHHHHHSSGLVPRGSHMASMTGGQQMGRGSEFLEVLFQ (containing a His_6_ tag and HRV 3C protease recognition site) at the N-terminus, expressed in *E. coli* using *p*ET-28a(+) as a vector
RfsMut8S: Removal of amino acid fusion sequence from HHMut8S by HRV 3C protease
his_6_-hrv-mut8S-p28: Plasmids constructed in this study to express HHMut8S using *p*ET-28a(+) as a vector

## Highlights

> A mutant was designed to form more salt bridges than the wild-type exo-inulinase.

> Rigidity of termini and flexibility of catalytic domain of the mutant were increased.

> Thermostability and low-temperature activity of the mutant were improved.

> Giving a new efficient way to overcome the activity–stability trade-off of enzymes.
